# Crystals at a Carrefour on the Way through the Phase Space: A Middle Path

**DOI:** 10.3390/molecules26061583

**Published:** 2021-03-13

**Authors:** Yury V. Torubaev, Ivan V. Skabitsky

**Affiliations:** Кurnakov Institute of General and Inorganic Chemistry, Russian Academy of Sciences, 119991 Moscow, Russia; skabitskiy@gmail.com

**Keywords:** non-covalent interactions, chalcogen bonding, polymorphism, supramolecular synthons, synthons evolution, synthon crossover, crystallization, structural landscape, organotellurium, crystal design, energy framework

## Abstract

Multiple supramolecular functionalities of cyclic α-alkoxy tellurium-trihalides (including Te---O, Te---X (X = Br, I) and Te---π(C=C) supramolecular synthons) afford rich crystal packing possibilities, which consequently results in polymorphism or Z’ > 1 crystal structures. Example of three crystal forms of cyclohexyl-ethoxy-tellurium-trihalides, one of which combines the packing of two others, affords a unique model to observe the *supramolecular synthon evolution* at the early stages of crystallization, when *crystals on the way* find themself at a carrefour between the evolutionally close routes, but fail to choose between two energetically close packing patterns, so taking the “middle path”, which incorporates both of them (and results in two crystallographically independent molecules). In general, this allows a better understanding of the existing structures, and an instrument to search for the new polymorphic forms.

## 1. Introduction

*“…the one parcel of phase space”* [1]. Due to the frequent occurrence of chalcogen bonding interactions (ChB) [2] at electrophilic Te centres and other non-covalent interactions (NCI) in organotellurium halides, their supramolecular chemistry is richer than their molecular one. The tendency towards non-covalent and hypervalent binding increases as we move down the VIA group and multiple variants for self-organization around the Te centre naturally lead to synthon polymorphism in some of them [3,4].

**Scheme 1:**
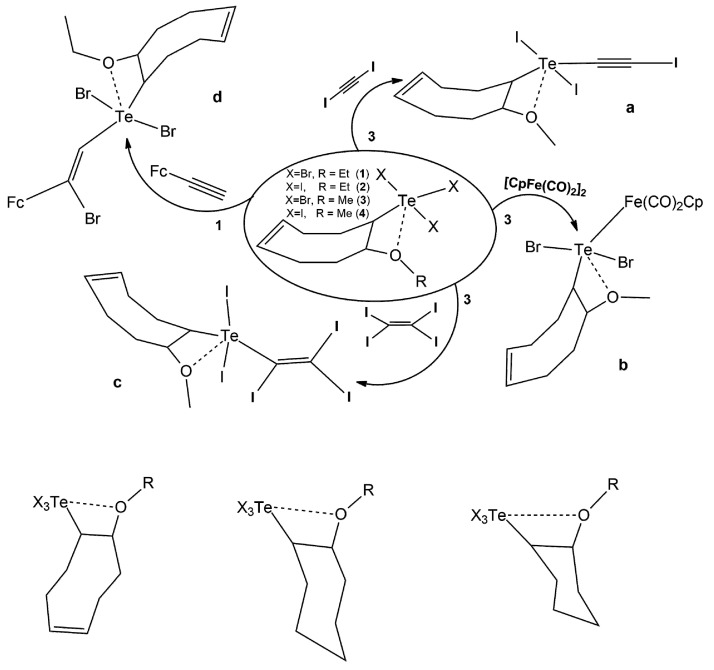
Interaction of cyclo-C_8_H_12_(OR)TeX_3_ with (**a**) diiodo-ethyne C_2_I_2_; (**b**) [CpFe(CO)_2_]_2_; (**c**) tetraiodo-ethene C_2_I_4_; (**d**) ferrocenyl-ethyne FcC_2_H.

This work arose in the course of our recent investigation of the unusually easy electrophilic addition of organo-tellurium trihalides to the C-C triple bond of ferrocenylacetylene [5], formal substitution of one of the bromides by iodoacetylide and triiodoethylenic fragments [6] and oxidative cleavage of the Fe-Fe bond in [CpFe(CO)_2_]_2_ [7] (see Scheme 1).

**Scheme 2:**
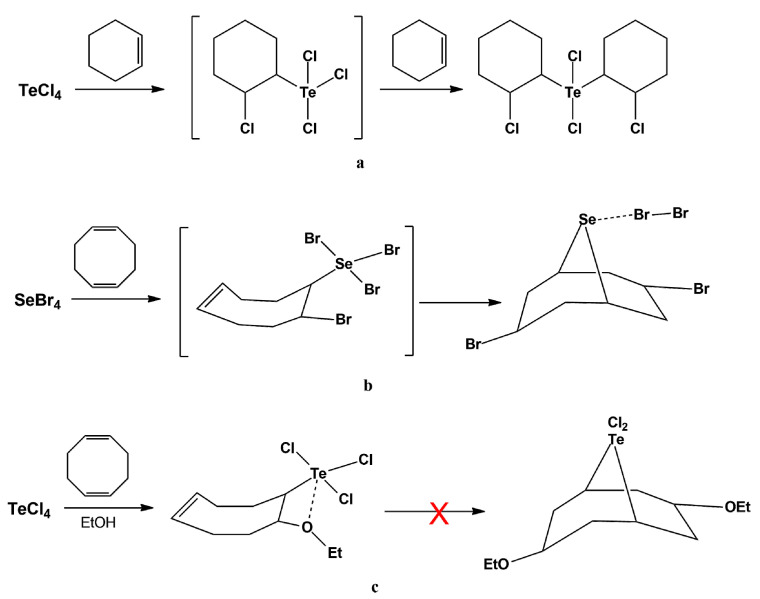
Interaction of (**a**) cyclo-hexene with TeCl_4_; cyclo-octa-1,5 diene with (**b**) SeBr_2_; (**c**) TeCl_4_.

In the context of such chemical reactivity of cyclo-C_8_H_12_(OR)TeX_3_, some cases of its inertness deserve special attention. For example, no further intra- or intermolecular reaction between Te and a double bond is observed, although SeBr_4_ and SCl_2_ interact with both double bonds of 1,5-cyclooctadiene (COD) to form bicyclic cyclooctane chalcogenides [8] or, say, TeCl_4_ interacts with an excess of cyclohexene resulting in cyclohex-TeCl_3_, which further attacks the double C=C bond of another cyclohexene molecule to form cyclohex_2_TeCl_2_ [9] (see Scheme 2).

This unusual structural and chemical behaviour of cyclic alkoxy-organotellurium trihalides suggested a closer look at their crystal structure, therefore, we undertook the XRD structural investigation of cyclo-C_8_H_12_(OR)TeX_3_ (R = Me, Et, X = Br, I), its cyclohexane α-alkoxy tellurium-trihalide congeners and a comparative analysis of the complex interplay between Te---O, Te---X (X = Br, I) and Te—π(C=C) chalcogen bonding in their solid-state structures.

## 2. Results and Discussion

### 2.1. Supramolecular Architectures of Cyclic Alkoxy-Organotellurium Trihalides

**Figure 1:**
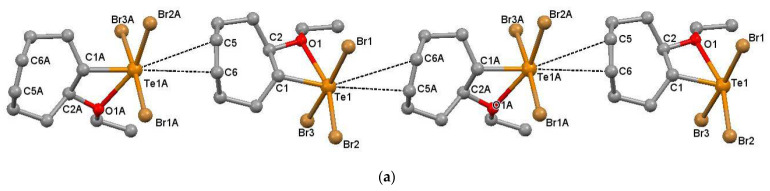

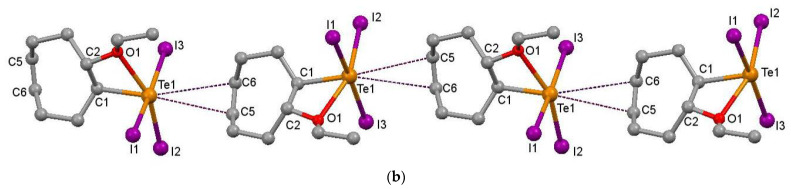
Fragments of the packing pattern of **1** (P21/c, Z’ = 2, (**a**)) and **2** (Pbca, Z’ = 1 (**b**)). Notice the (**a**) 1D chain of altering *R,R* and *S,S* molecules in **1** and the (**b**) 1D chain of S,S chain of **2**. Hydrogen atoms are omitted for clarity. Selected distances in **1** (Ǻ): (intramolecular) Br(2)-Te(1) 2.5224(5), Br(3)-Te(1) 2.6632(6), Br(1)-Te(1) 2.6826(5), O(1)-Te(1) 2.389(3); (intermolecular): Te(1A)-C(6) 3.430(3), Te(1A)-C(5) 3.648(4), Te(1)-C(6A) 3.830(4), Te(1)-C(5A) 3.435(3). Selected distances in **2** (**b**) (Ǻ): (intermolecular) Te1---C5 3.548(5), Te1---C6 3.592(5).

**Figure 2:**
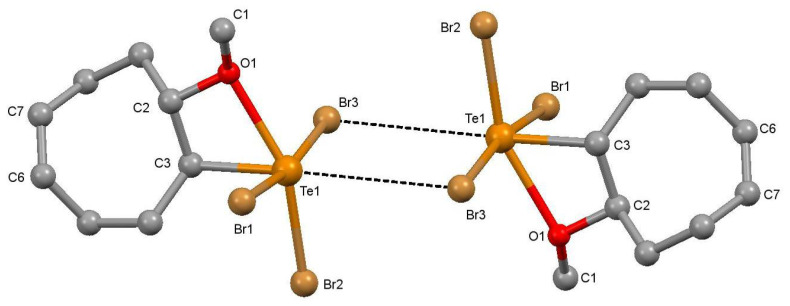
Te---(µ-Br) centrosymmetric dimers of **3**. Hydrogen atoms are omitted for clarity. Selected distances (Ǻ): (intramolecular); Br(1)-Te(1) 2.6562(5), Te(1)-Br(2) 2.5121(7), Te(1)-Br(3) 2.6880(5), Te(1)-O(1) 2.416(3), (intermolecular) Br(3)-Te(1) 3.7389(6).

Analysis of the crystal structure of the 8-alkoxy-4-cyclooctenyl-tellurium tribromides **1** and **3** (see Scheme 1) demonstrated that, despite the fact that they are close homologues (R = Et (1) and Me (3)), they provide different self-assembly patterns in solid-state. In particular, ethoxy-homologues C_8_H_12_(TeX_3_)OEt (R = Et, X = Br (**1**), I (**2**)) are assembled into 1-D chains stabilized by Te—π(C=C) ChBs (3.479–3.577 Å) (see Figure 1a), while their chloro- (X = Cl, R = Et [10] and methoxy- (R = Me X = Br (**3**), I (**4**) congeners, form dimeric associates [11,12,13], stabilized by two Te---X ChBs (see Figure 2). The tellurium center in **1**–**2** can be described as a typical octahedral [14] (see Figure 1), with the sixth position of the octahedron occupied by a weakly associated C=C double bond.

In an electron-withdrawing environment, the electrophilic Te atom tends to form Te---π(C≡C)_acetylene_ chalcogen bonds (ChB). Like Te---π-aryl ChBs [15], the Te---π-acetylene ChBs are well documented [16], except for, to the best of our knowledge and after a Cambridge Structural Database (CSD) search, the Te---π(C=C)_alkene_. Chalcogen bonding found in **1** and **2** is unprecedented.

Taking into account the existence of centrosymmetric dimeric associates of C_8_H_12_(TeCl_3_)OEt [10], we can assume the existence of the corresponding polymorphs of **1** and **2**. Although our attempts to crystallize them were not successful, the expected Te---X centrosymmetric dimers were found in their methoxy congeners: the isomorphic pair of *cyclo*-C_8_H_12_TeBr_3_(OMe) (**3**) and *cyclo*-C_8_H_12_TeI_3_(OMe) (**4**) (see Figure 2).

**Figure 3:**
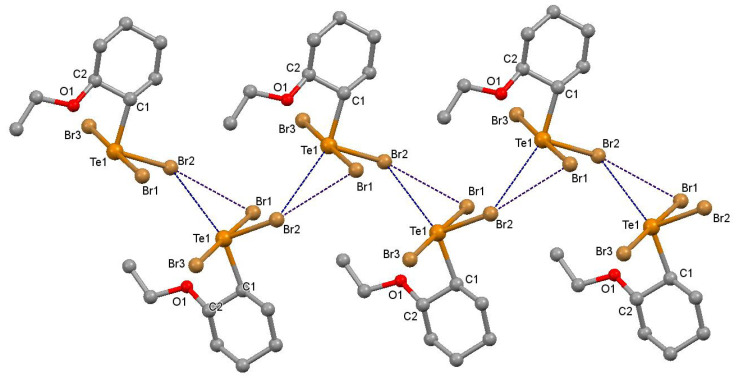
Fragment of packing in the crystal of **6** (*P*2_1_/*n*) showing the Te---I ChB and Br---Br XBs stabilized catemer chain. Hydrogen atoms are omitted for clarity. Selected distances (Ǻ): (intramolecular) Te1-Br1 2.6749(4), Te1-Br3 2.6271(4), Te1-Br2 2.4800(5), Te1-O1 2.716(2); (intermolecular); Te1-Br2 3.9027(5), Br2-Br1 3.5956(5).

**Figure 4:**
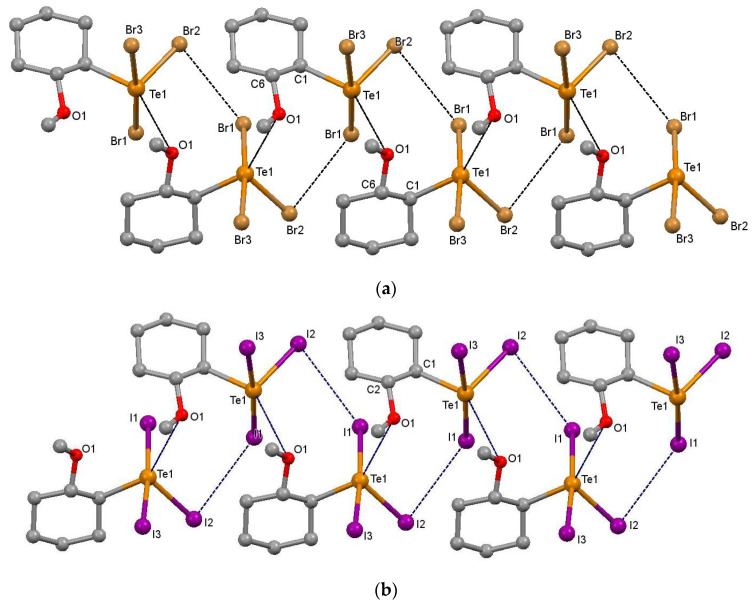
Fragment of packing in the isostructural crystal of **7** (**a**) and **8** (**b**). Showing the Te---O ChB and X---X (X = Br, I) XBs stabilized catemer chain. Hydrogen atoms are omitted for clarity. Selected distances (Å) in **7**: (intramolecular): Te1-O1 2.826, Te1-Br3 2.679(2), Te1-Br1 2.639(2), Te1-Br2 2.491(2); (intermolecular) Te1-O1 3.422(9), Br2-Br1 3.778(2); Selected distances (Å) in **8** (intramolecular): Te1-O1 2.915(6), I1-Te1 2.9205(6), Te1-I3 2.8958(7), Te1-I2 2.7165(7) (intermolecular): Te1-O1 3.604(5), I2-I1 3.9888(7). Notice the obscene of Te---halogen intermolecular interactions.

Under the same conditions as for **1**–**4**, but using the cyclohexene instead of COD, we obtained a series of 2-alkoxy-cyclohexyl-tellurium trihalides *cyclo*-C_6_H_10_TeX_3_(OR) (X = I, R = Et (**5**), X = Br, R = Et (**6**), X = Br, R = Me (**7**), X = I, R = Me (**8**). The synthesis and ^13^C/^1^H NMR characterization of tribromides **6**, **7** was reported earlier [17], but the trichloride *cyclo*-C_6_H_10_TeCl_3_(OEt) has been the only structurally characterized member of this family until now [18]. Our SC-XRD investigation **5**–**8** demonstrated a noticeable lengthening of intramolecular Te---O distance in **5**–**8** (~2.7–2.8 Ǻ) compared to ~2.4 Å in **1** and **2** (see Figure 1, Figure 2, Figure 3 and Figure 4). This lengthening of the distance between adjacent substituents in the six-member ring is a natural consequence of the rigid torsion angles in the six-membered ring, compared to the more flexible seven- and eight-membered rings (see Table S1 and Scheme S1).

Analysis of the molecular geometry around the Te center in this series suggests that it is mainly the steric rather than the electronic motif that drives the TeX_3_ and OEt functions apart from each other so that in the seven-membered 2-ethoxy-cycloheptyl-tellurium tribromide [19] (see Table S1) the Te---O distance (2.492 Å) is exactly between C_6_ and C_8_ derivatives. This rather mechanical weakening of the intramolecular Te---O bonding in **5**–**8** leads to a switching of electrophilic Te from the *intra*-molecular Te---O interaction with the methoxy-oxygen atom in **1**–**3** to the *inter*-molecular Te---O bonding in isomorphic **7** (3.427 Å) and **8** (3.604 (8) Å) (see Figure 4).

Formal substitution of Et (in **6**) for a more compact Me group (in **7**) can reduce the shielding of the alkoxy-oxygen atom and allow closer interaction between the molecules, which leads to the stabilization of the catemer chain by Te---O ChBs. Analysis of the bonding angles around the Te---O ChB and X---X (X = Br (**7**), I (**8**)) XB interactions in the catemer chains shows that they are genuine (type II) σ-hole bonds.

### 2.2. Polymorphism of Cyclo-C_6_H_10_(OEt)TeI_3_

**Figure 5:**
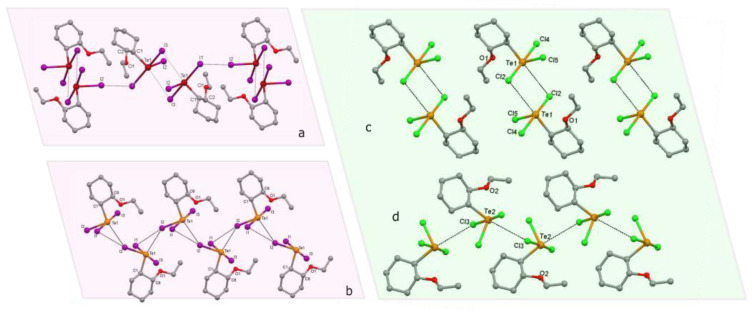
Fragment of packing of **5a** (**a**), **5b** (**b**) and C_6_H_10_(OEt)TeCl_3_ (WEQTUH (**c**,**d**) showing the Te---X ChB stabilized centrosymmetric dimers in in **5a** (**a**) and C_6_H_10_(OEt)TeCl_3_ (**c**); and Te---X ChB stabilized catemer chain in **5b** (**a**) and C_6_H_10_(OEt)TeCl_3_ (**d**). Hydrogen atoms are omitted for clarity. Selected distances (Ǻ) in **5a** (**a**): (intramolecular) O1-Te1 2.796(4), I2-Te1 2.7243(5), I1-Te1 2.9664(5), I3-Te1 2.8520(5); (intermolecular); Te1-I3 3.9199(6); Selected distances (Ǻ) in **5b** (**b**): (intramolecular) I2-Te1 2.699(1), Te1-I3 2.717(2), Te1 I1 2.869(1), Te1 O1 2.775(9); (intermolecular); Te1-I2 3.902(1), I1-I2 3.493(2).

Crystallization of a saturated solution of **5** from DCM/hexane (3:1 mixture) yields the crystals that are isostructural with **6** and therefore are packed in the centrosymmetric Te---I ChB dimers (**5a** see Figure 5a). Further crystallization of the filtered mother liquor resulted in another polymorphic form of **5**, build of catemeric chains, stabilized by Te---I ChB (**5b,** see Figure 5b). Surprisingly, in the crystal of C_6_H_10_(OEt)TeCl_3_ (WEQTUH [18], which is the chloride congener of **5**), we noticed both dimeric [S,S RTeCl_2_(µ-Cl)]_2_ and catemer [R,R RTeCl_2_(µ-Cl)] architectures are simultaneously and independently present in one (and so far the only) crystal form (see Figure 5c,d).

**Figure 6:**
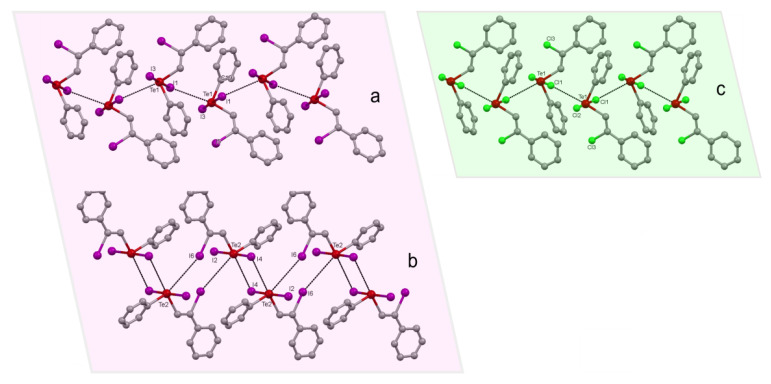
Fragment of packing of (Z)-2-iodo-2-phenylvinyl)-phenyl-tellurium diiodide (Z’ = 2, **a**,**b**) and (Z)-2-chloro-2-phenylvinyl)-phenyl-tellurium dichloride (Z’ = 1, **c**), showing the parallel chains of Te---I ChB stabilized catemer (**a**) and centrosymmetric dimers (**b**) in Ph(I)C=C(H)TeI_2_Ph (CUCQEX) and Te---Cl ChB stabilized catemer chains in in Ph(Cl)C=C(H)TeCl_2_Ph (GOKFAO). Hydrogen atoms are omitted for clarity.

**Figure 7:**
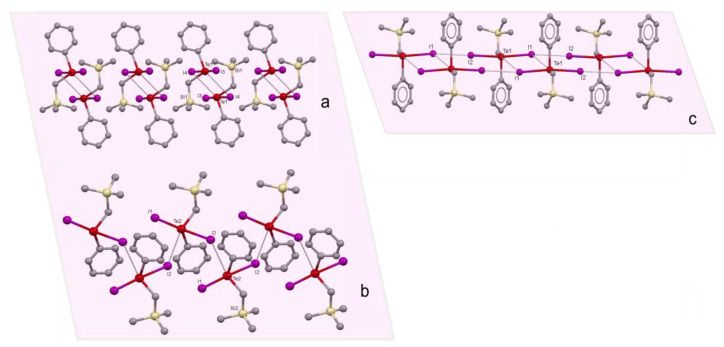
Fragment of packing of two polymorphic forms of phenyl-(trimethylsilylmethyl)-tellurium diiodide: (**a**,**b**)—dimer/catemer parallel chains (ISALOE01, *C2/c*, Z’ = 2) and (**c**)—dimeric (ISALOE, *P-1*, Z’ = 1) (**c**), showing the parallel chains of Te---I ChB stabilized centrosymmetric dimers (**a**) and catemer (**b**) in *C2/c* form and only associated dimers in *P-1* form (**c**). Hydrogen atoms are omitted for clarity. Notice that dimeric chains (**a**) and (**c**) have a different packing pattern.

**Figure 8:**
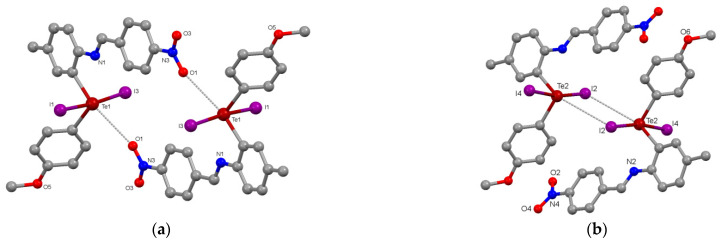
Two supramolecular synthons (**a**) Te---O and (**b**) [Te---Cl]_2_ in the crystal of di-iodo-(2-(4-nitrobenzylideneamino)-5-methylphenyl)-(4-methoxyphenyl)-tellurium (TARGAV).

This suggests that special attention should be paid to the packing patterns of each independent molecule in the crystals with Z’ > 1, in the light of rationalizing the existing, as well as predicting possible polymorphism. With this in mind, we searched through the Z’ ≥ 2 RTeX_n_ (*n* = 1,2) structures deposited in CSD with the intention of identifying those that have two independent molecules and belong to two different packing patterns. Our surface CSD screening for the Z’ ≥ 2 was limited to organotellurium halides to be consistent with the main compounds of this work. Below are some of the most illustrative and relevant examples of the crystals that have two independent molecules, each of which belongs to a different type of packing pattern (see Figure 6, Figure 7 and Figure 8). The first Z’ = 2 crystal to mention is (Z)-2-iodo-2-phenylvinyl)-phenyl-tellurium diiodide (CUCQEX, see Figure 5)—a product of *anti*-Markovnikov electrophilic addition of PhTeI_3_ to phenylacetylene (PhC_2_H). Overlooked in our original communication in 2008 [20], like *cyclo*-C_6_H_10_(OEt)TeCl_3,_ it has both catemer chain (see Figure 5a) and dimeric associates (see Figure 5b) present in parallel in one (and yet the only) crystal form. Its cognate chloride (GOKFAO [21]) Z’ = 1 has only the catemer chains (see Figure 6c). It is noteworthy that these iodide and chloride catemer chains, belonging to different crystals, are quite identical, and we can assume the existence of a centrosymmetric dimeric form of chloride.

We noticed similar parallel dimer/catemer chains (see Figure 7a,b) vs. only dimeric crystal form (see Figure 7c) in a polymorphic pair of di-iodo-phenyl-(trimethylsilylmethyl)-tellurium (ISALOE01/ISALOE [22]). Unlike this, discrete dimers in Z’ = 2 (Figure 7a) and I---I associated dimers in Z’ = 1 (see Figure 7c) form different chains.

Not all of these tellurium halides with Z′ = 2 are characterized by the interplay of Te---halide dimer/catemer patterns. For example, in the crystal of diiodo-(2-(4-nitrobenzylideneamino)-5-methylphenyl)-(4-methoxyphenyl)-tellurium (TARGAV. P-1, Z’ = 2, see Figure 8a,b), the supramolecular synthon crossover [23] between [Te---I] and [Te-O=NO] resulted in two corresponding dimeric associates [24].

This is by no means an exclusive feature of telluro-halides, and a similar phenomenon was noted in the polymorph landscape of 1,1,4,4-tetraphenyl-1,3-butadiene [25]. Therefore, we can expect that new experimental data combined with deeper and more extensive CSD analysis, will definitely yield more Z’ > 2 “midway” crystals, which will advance our understanding of the crystallization mechanism and the prediction of new polymorphic forms.

### 2.3. Energy Frameworks

**Figure 9:**
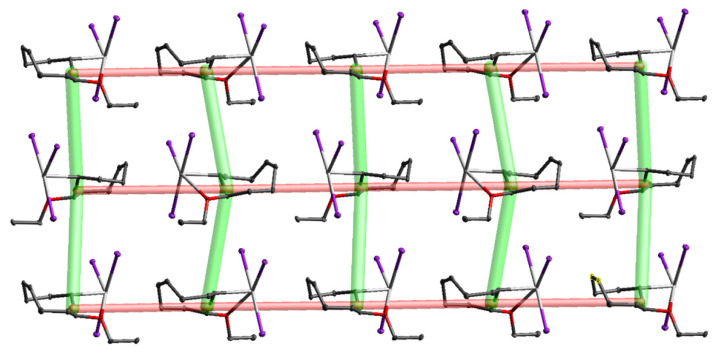
The energy framework of **2**. The electrostatic component is represented by a red cylinder (cutoff 30 kJ/mol), dispersion—by a green cylinder (Cutoff 35 kJ/mol). Notice the electrostatically stabilized chains, linked with one another by dispersion (van der Waals) interactions.

It is important to note that the chains and centrosymmetric dimers discussed for **1**–**8** are not just the visually observed patterns, the existence of which is only confirmed by short contacts, but correspond to the shape and geometry or energy framework of the corresponding crystal. The energy framework is the visualization of intermolecular interaction energies between pairs of molecules, presented in the form of cylinders joining the centroids of pairs of molecules (with a cylinder radius proportional to the magnitude of the interaction energy) [26]. They are an effective tool for visualizing the supramolecular architecture [27] or even supramolecular reactions [28,29,30,31], considering the molecular crystal as a whole. For example, the total energy framework of **5a** and **8** at the maximum cutoff (i.e., the strongest intermolecular interactions) matches the close contact pattern (see Figures S2–S4). However, for most of the remaining structures, not the total energy, but its electrostatic component framework has the same chain geometry as suggested by the short contacts pattern (see Figure 9, Figures S1 and S3).

Intermolecular interaction energies (CE-B3LYP DGDZVP) used for the energy framework for 2 were also evaluated using PBE0 and (DLPNO-CCSD(T) with def2 triple-zeta basis sets and demonstrated a good agreement (see Table S2 for details). It worth noting that the total energy of intermolecular interactions in a neutral molecular crystal is contributed mostly by electrostatic and dispersion components, and electrostatic interactions, in turn, fall-off as a minus two power of distance, while dispersion falls-off as a minus six power of distance [32]. This leads us to suggest that chalcogen bonding (which is dominated by electrostatic) may have a stronger orienting effect at the earliest stages of nucleation, pre-organizing the molecules into the chains (according to Kitaigorodsky Aufbau Principle, KAP [33]), which further, according to the same KAP, are associated into the layers and so on up to the 3D crystal structure. This structure-directing influence of specific chemical interactions (ChB, XB) can be noticed in a resulting crystal structure as the predominantly electrostatically stabilized architectures.

## 3. Materials and Methods

All reactions and manipulations were performed using standard Schlenk techniques under an inert atmosphere of pure nitrogen or argon. Solvents were purified, dried and distilled in nitrogen or argon atmosphere prior to use. Commercial reagents (cyclohexene, 1,5-cyclooctadiene) were distilled before use. C_8_H_12_TeBr_3_OR and C_6_H_10_TeBr_3_OR were prepared following the reported procedures [7,10]. Tri-iodides **2**, **4**, **5**, **8** were prepared by Finkelstein reaction of the respective bromides with KI in acetone as described in [7].

### 3.1. X-ray Crystallography

Suitable X-ray quality crystals of **1**–**2** and **5**–**8** were obtained directly during preparation (see the synthetic part for details). A Bruker APEX II CCD area detector diffractometer equipped with a low-temperature attachment was used for the cell determination and intensity data collection for compounds **1**–**2** and **5**–**8**. Structures **1**–**2** and **5**–**8** were solved by direct methods and refined by means of the least squares method for F^2^ in anisotropic (isotropic for H atoms) approximation using Olex2 and SHELXTL software [34,35]. Positions of H atoms were calculated geometrically. Appropriate empirical absorption corrections were made using the programs SADABS. Atomic coordinates and other structural parameters of **1**–**2** and **5**–**8** have been deposited with the Cambridge Crystallographic Data Centre CCDC 760,039 (**1**), CCDC 760,040 (**2**), CCDC 2,062,201 (**5a**), CCDC 2,062,200 (**5b**), CCDC 2,062,202 (**6**), CCDC 2,062,203 (**7**) and CCDC 2,062,204 (**8**), which also contains the supplementary crystallographic data for this paper.

### 3.2. Computational Details

Intermolecular interaction energy calculation and subsequent Hirshfield surfaces [36] and energy frameworks generation were performed using Crystal Explorer 17.5 (TONTO, B3LYP-DGDZVP) [26] for all unique molecular pairs in the first coordination sphere of a molecule (4.8 Å), using experimental crystal geometries.

Pairwise interaction energies for XRD dimer geometries provided by Crystal Explorer were also evaluated for 2 using the hybrid functional PBE0 [37] and domain based local pair natural orbital coupled cluster theory with single-, double-, and perturbative triple excitations (DLPNO-CCSD(T) [38] implemented in ORCA 4.1.2 program package [39], def2 triple-zeta basis sets [40] and appropriate correlation fitting basis sets [41] were used for all calculations. Basis set superposition error was avoided by using dimer basis for monomer energy evaluation. TightPNO keyword was used for accuracy control of DLNO calculation [42]. Dispersion correction with Becke–Johnson damping (D3BJ) [43,44] was used for energies calculated with DFT. This evaluation demonstrated a good agreement between CE-B3LYP and higher level computations (see Table S2 for details).

## 4. Conclusions

The electrophilic tellurium atom in organoalkoxy-tellurium trihalides has a distinct tendency to form intermolecular Te---halide, Te---O, Te---π(C=C) chalcogen bonds (ChB). In some cases, the formation *inter-*molecular Te---O ChBs may compensate for the weakening of *intra*-molecular Te---O ChBs. This complex interplay of Te-centered ChBs and thermodynamic/lattice symmetry factors provides a complex structural landscape with several close-by local minima, resulting in polymorphism and different packing patterns of organotellurium halides. The slightest changes in the chemical structure and/or crystallization conditions can shift the balance towards one of these multiple variants of packing patterns: Te—X dimers, chains, Te---O chains and their combinations. In this context, the case of catemer chains and centrosymmetric dimers, both observed independently in the same lattice of C_6_H_10_TeCl_3_OEt (WEQTUH [18] see Figure 5) provides convincing evidence that crystallographically independent fragments are *fossil relics* of early stages of the crystallization process [45], when two precursors (i.e., supramolecular synthons [46]) of two different polymorphic forms can coexist in one crystal.

This is typical not only for organotellurium halides—they are just good examples of compounds capable of forming various combinations of energetically close σ-hole interactions. Previously, it was shown that *hydrogen bonding potential slightly increases a molecule’s likelihood of being polymorphic* [47]; therefore, other types of weak σ-hole intermolecular interactions can also increase the chances of polymorphism. However, unlike hydrogen bonds, the latter (XBs. ChBs) are more directional and specific [48,49,50] and, therefore, can make a polymorphic pattern more predictable. In this work, we observed the supramolecular *synthons evolution* [51] at the early stages of crystallization, when crystal *on the way* [52] through the *phase space* [1], finds itself at a carrefour between the evolutionally close routes, but fails to choose one, therefore taking the “middle path”, which incorporates both packing patterns (and results in two crystallographically independent molecules). We expect that further analysis of packing patterns of each independent molecule in the single-component crystal structures with Z’ = 2, with particular attention to polymorphs, can provide more important examples for later understanding of the early stages of crystal genesis.

## Data Availability

The data presented in this study are available in this article.

## Supplementary Materials

The following are available online, Figure S1: The energy framework of 2. (a) Electrostatic component is represented by a red cylinder (cut-off 30 kJ/mol); dispersion by a green cylinder (cut-off 35 kJ/mol). (b) Total intermolecular (blue cylinder, 30 kJ/moll cut-off); Figure S2: The energy framework of 5a. Electrostatic component is represented by red cylinder (cut-off 43 kJ/mol); dispersion by green cylinder (cut-off 25 kJ/mol). (b) Total intermolecular (blue cylinder, 24 kJ/moll cut-off). Figure S3: The energy framework of 5b. (a-b) Electrostatic component is represented by red cylinder (cut-off 32 kJ/mol); (b) dispersion by green cylinder (cut-off 32 kJ/mol). (c) total intermolecular (blue cylinder, 20 kJ/moll cut-off). Figure S4: The energy framework of 8. Electrostatic component is represented by red cylinder (cut-off 38 kJ/mol); dispersion by green cylinder (cut-off 20 kJ/mol). (b) Total intermolecular (blue cylinder, 30 kJ/moll cut-off); Table S1: Intramolecular Te---O distances in 1,2—alkoxy cyclic tellurium trihalides; Table S2: Interaction Energies (kJ/mol) ) for the 5 Å cluster of 2 (see Figure S5); Scheme S1: Elongation of the Te---O distance in a row C_8_ > C_7>_ C_6_. Notice the slight variation in the angles around the C-C bond and significant increase of the Te-C-C-O torsion changes as we move from the six- to eight-member ring. This is typical for cycloalkanes since the increase of the chain length facilitates the torsion.

## Sample Availability

Not available.
