# Supplementary material for: Crystals at a Carrefour on the Way through the Phase Space: A Middle Path
Source: Molecules. 2021 Mar 13;26(6):1583. doi: 10.3390/molecules26061583 (PMC7998266; doi:10.3390/molecules26061583)
Supplement: Supplementary file 1 [file molecules-26-01583-s001.pdf]

Electronic Supplementary Information

Crystals at a Carrefour on the Way Through the Phase Space: A Middle Path.

Yu.V. Torubaev and I.V. Skabitsky.

N.S. Kurnakov Institute of General and Inorganic Chemistry of Russian Academy of Sciences, Moscow.

Table S1. Intramolecular Te---O distances in 1,2—alkoxy cyclic tellurium trihalides.

		X=Cl	X=Br	X=I
cyclo-C_6 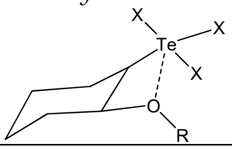	Te----O_{Et}	2.643-2.657 [1]	2.716	2.775
	Te---O_{Me}	n/a	2.826	n/a
cyclo-C_7 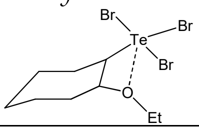	Te---O_{Et}	n/a	2.492 [2] 19.0	n/a
cyclo-C_8 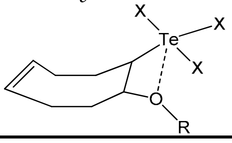	Te---O_{Et}	2.419 [3]	2.389(3) - 2.403(3) 15.15	2.461(3)

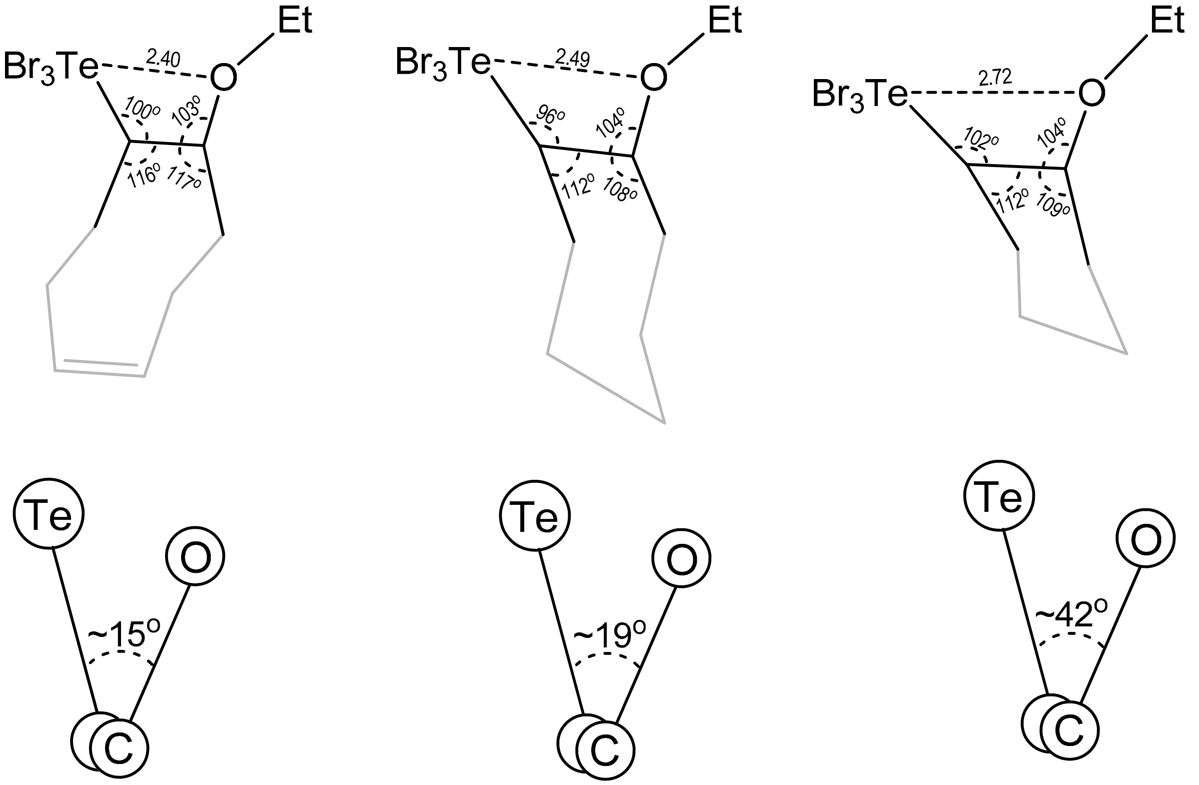

Scheme S1. Elongation of the Te---O distance in a row $C_8 > C_7 > C_6$. Notice the slight variation in the angles around the C-C bond and significant increase of the Te-C-C-O torsion changes as we move from the six- to eight-member ring. This is typical for cycloalkanes since the increase of the chain length facilitates the torsion.

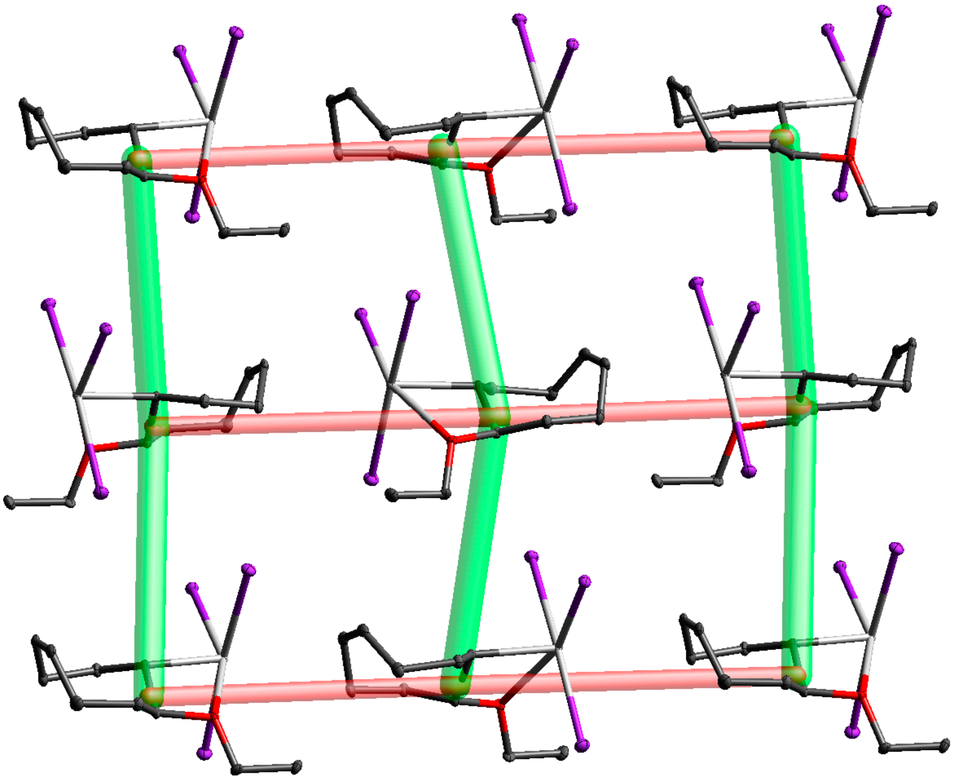

a

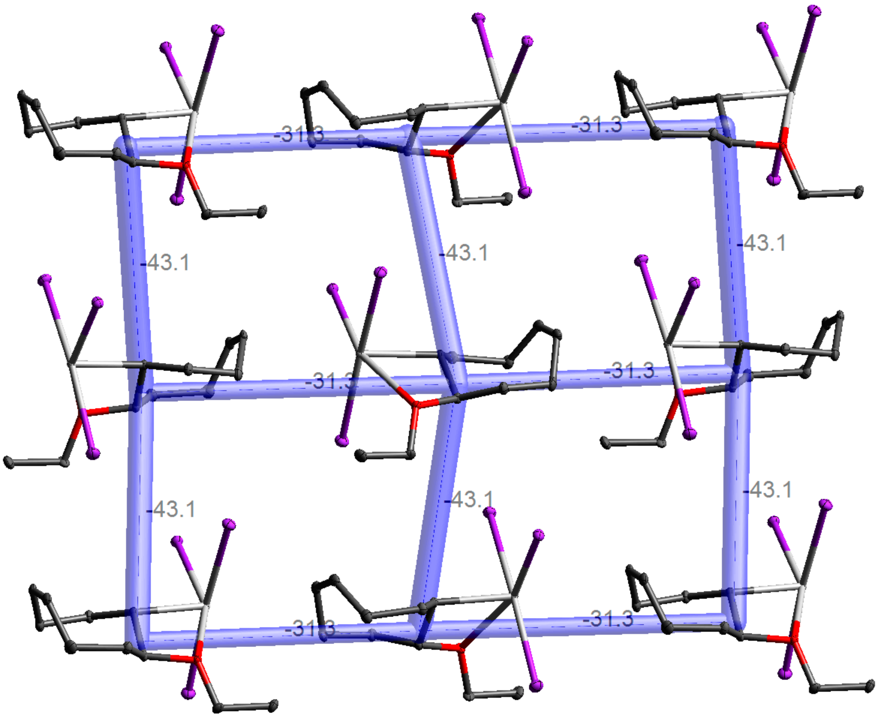

b

Figure S1. The energy framework of **2**. (a) Electrostatic component is represented by a red cylinder (cut-off 30 kJ/mol); dispersion by a green cylinder (cut-off 35 kJ/mol). (b) Total intermolecular (blue cylinder, 30 kJ/moll cut-off).

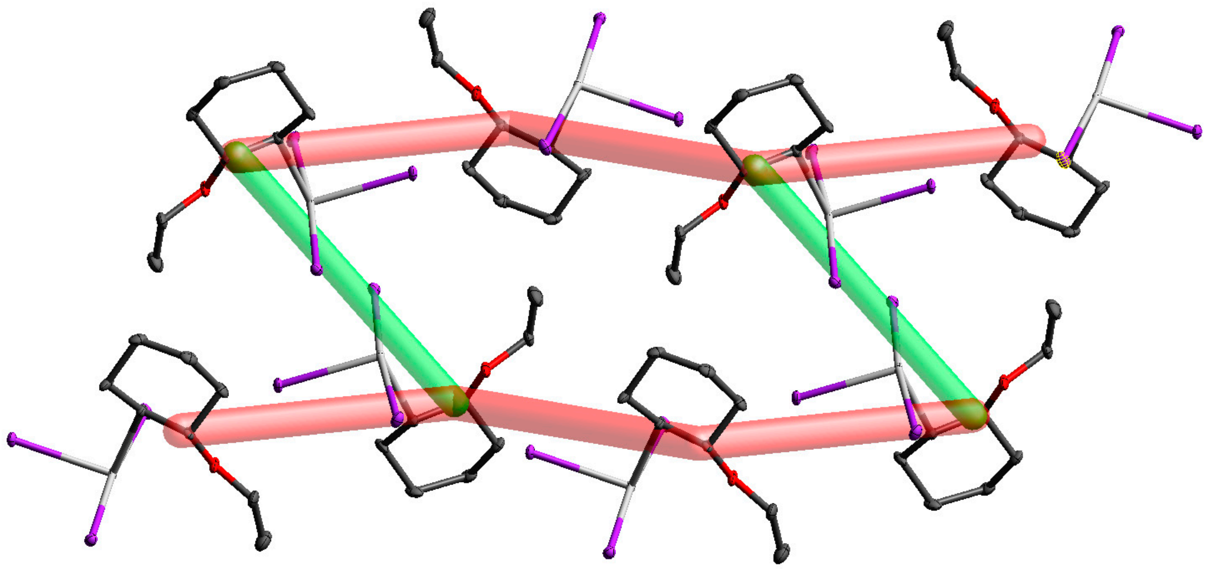

a

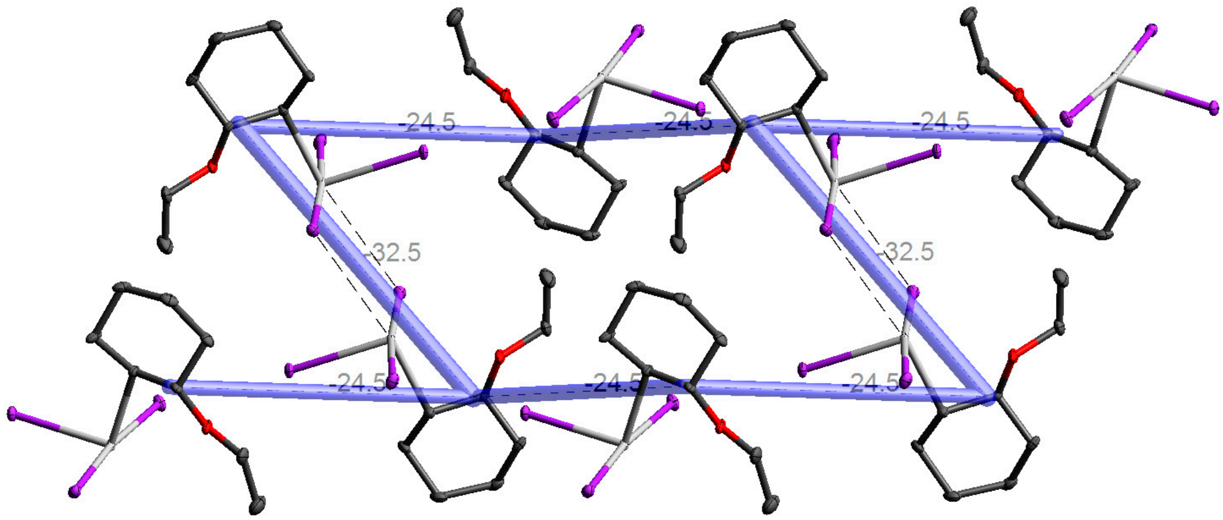

b

Figure S2. The energy framework of **5a**. Electrostatic component is represented by red cylinder (cut-off 43 kJ/mol); dispersion by green cylinder (cut-off 25 kJ/mol). (b) Total intermolecular (blue cylinder, 24 kJ/moll cut-off).

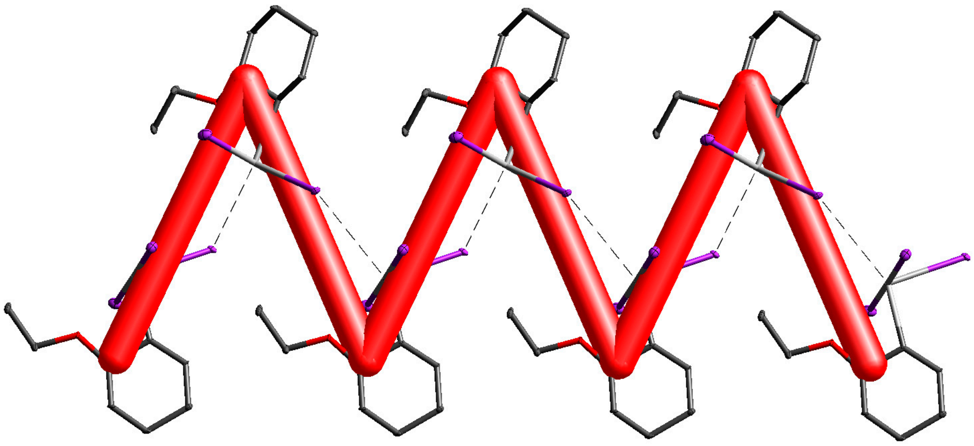

a

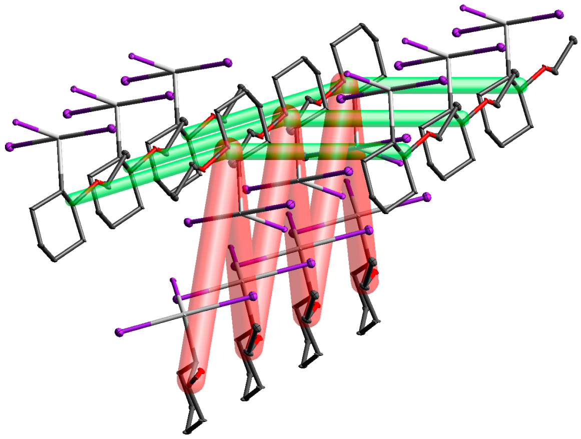

b

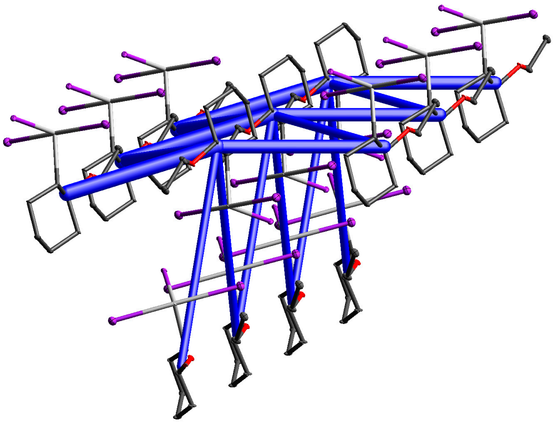

b

Figure S3. The energy framework of **5b**. (a-b) Electrostatic component is represented by red cylinder (cut-off 32 kJ/mol); (b) dispersion by green cylinder (cut-off 32 kJ/mol). (c) total intermolecular (blue cylinder, 20 kJ/moll cut-off).

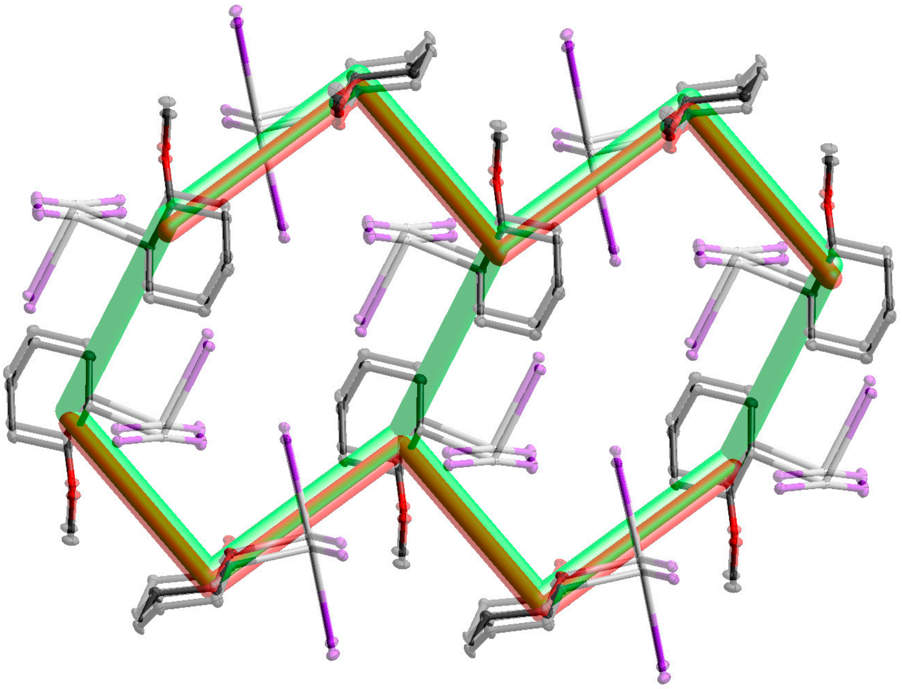

a

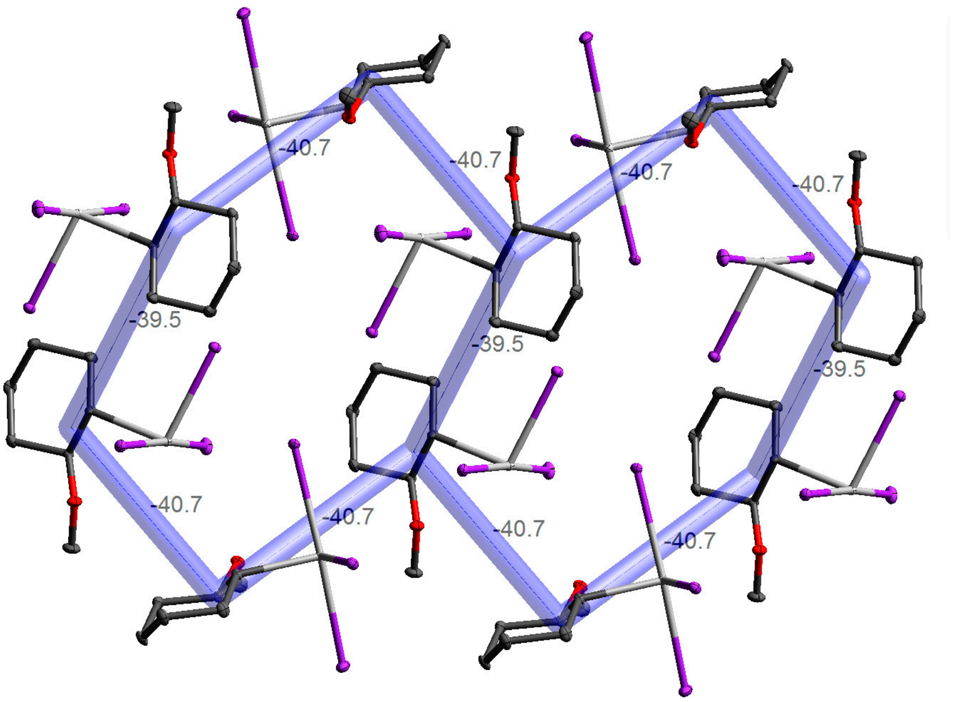

b

Figure S4. The energy framework of **8**. Electrostatic component is represented by red cylinder (cut-off 38 kJ/mol); dispersion by green cylinder (cut-off 20 kJ/mol). (b) Total intermolecular (blue cylinder, 30 kJ/moll cut-off).

Table S2. Interaction Energies (kJ/mol)) for the 5 Å cluster of **2** (see Fig. S5).

R is the distance between molecular centroids (mean atomic position) in Å.

		**CE-B3LYP DGDZVP**					PBE0/def2-TZVP	DLPNO-CCSD(T)/ def2-TZVP
**Symop**	**R**	**E_ele**	**E_pol**	E_dis	E_rep	E_tot		
x+1/2, -y+1/2, -z	7.24	-22.5	-5.5	-54.9	52.7	**-43.1**	-46.8	-33.8
-x, -y, -z	8.48	-22.9	-6.4	-50.8	43.8	**-46.0**	-46.7	-36.5
-x, y+1/2, -z+1/2	13.31	1.2	-0.2	-2.3	0.0	**-0.9**	-1.7	-1.2
-x+1/2, y+1/2, z	10.44	-6.7	-0.9	-11.3	12.8	**-9.7**	-10.5	-7.8
-x+1/2, y+1/2, z	9.01	-22.7	-0.8	-8.0	57.0	**3.6**	-11.3	-5.6
-x, -y, -z	6.79	-22.4	-2.8	-44.3	42.8	**-37.9**	-40.7	-30.0
x, -y+1/2, z+1/2	8.58	-28.7	-1.9	-38.6	55.3	**-31.3**	-34.3	-23.0
x+1/2, y, -z+1/2	8.83	-3.5	-1.1	-10.3	8.2	**-8.3**	-10.1	-7.1

Total energies are the sum of the four energy components, scaled appropriately [4].

Scale factors:

**k_ele**	**k_pol**	**k_disp**	**k_rep**
1.057	0.740	0.871	0.618

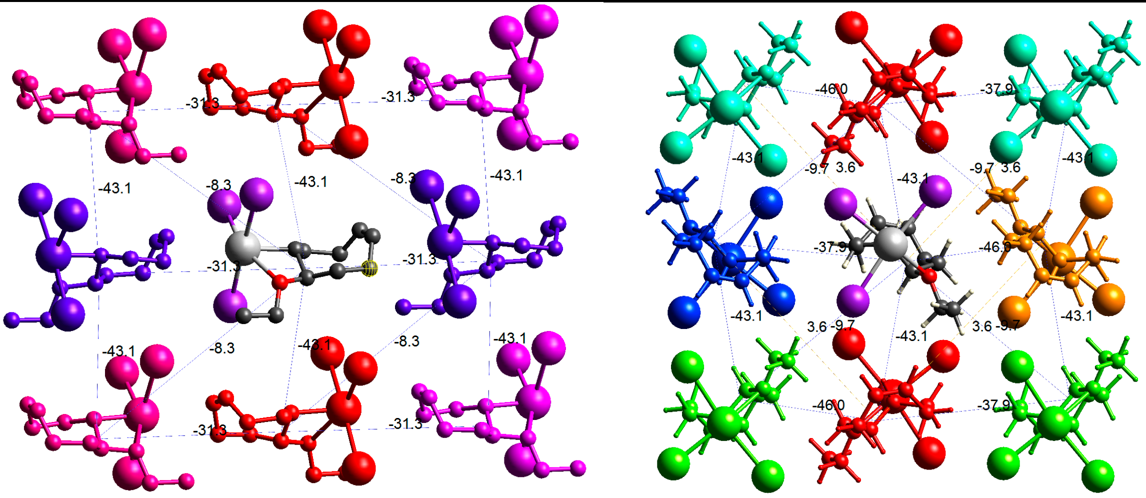

a **b**

Figure S5. Intermolecular interactions in 5 Å cluster of **2** . Energies are given in kJ/mol (CE-B3LYP DGDZVP, see Table S2).

Please see our paper in *Zeitschrift für Kristallographie - Crystalline Materials (2020)* [5] for more details on evaluation of Crystal Explorer B3LYP-DGDZVP data with DLPNO-CCSD(T)/def2-TZVP.

References

1. Dakternieks, D.; O'Connell, J.; Tiekink, E. R. T., Synthesis and crystal structures of the monomeric organotellurium(IV) trihalides: trans-2-ethoxy-cyclohexyl-tellurium(IV) trichloride, trichloro(2-chlorobicyclo[2.2.1]hept-7-yl)-λ4-tellurane, and mesityltellurium(IV) tribromide. *Journal of Organometallic Chemistry* **2000,** *598* (1), 49-54 %U https://linkinghub.elsevier.com/retrieve/pii/S0022328X99006695.
2. McAdam, C. J.; Cameron, S. A.; Hanton, L. R.; Manning, A. R.; Moratti, S. C.; Simpson, J., Probing CH-π(alkyne) interactions in a series of ethynylferrocenes. *CrystEngComm* **2012,** *14* (13).
3. Bergman, J.; Engman, L., Tellurium in organic synthesis. *Journal of Organometallic Chemistry* **1979,** *181* (2), 335-347 %U https://linkinghub.elsevier.com/retrieve/pii/S0022328X00828260.
4. Mackenzie, C. F.; Spackman, P. R.; Jayatilaka, D.; Spackman, M. A., CrystalExplorer model energies and energy frameworks: extension to metal coordination compounds, organic salts, solvates and open-shell systems. *IUCrJ* **2017,** *4* (Pt 5), 575-587.

5 Torubaev, Y. V.; Skabitsky, I. V., Halogen bonding in crystals of free 1,2-diiodo-ethene (C2H2I2) and its π-complex [CpMn(CO)2](π-C2H2I2). *Zeitschrift für Kristallographie - Crystalline Materials* **2020,** *235* (12), 599-607.
